# Lymphatic filariasis in Brazil: epidemiological situation and outlook for elimination

**DOI:** 10.1186/1756-3305-5-272

**Published:** 2012-11-26

**Authors:** Gilberto Fontes, Anderson Brandão Leite, Ana Rachel Vasconcelos de Lima, Helen Freitas, John Patrick Ehrenberg, Eliana Maria Mauricio da Rocha

**Affiliations:** 1 Universidade Federal de Alagoas, Maceió, Alagoas, Brazil; 2 Universidade Federal de São João del Rei, Campus Centro Oeste, Rua Sebastião Gonçalves Coelho, 400, Divinópolis, Minas Gerais, 35501-296, Brazil; 3 Secretaria de Vigilância em Saúde do Ministério da Saúde, Brasilia, Brazil; 4 World Health Organization (WHO – WPRO), Western Pacific Regional Office, Manila, Philippines

**Keywords:** Lymphatic filariasis, Epidemiology, *Wuchereria bancrofti*, Bancroftian filariasis, Brazil

## Abstract

Since the World Health Assembly’s (Resolution WHA 50.29, 1997) call for the elimination of lymphatic filariasis by the year 2020, most of the endemic countries identified have established programmes to meet this objective. In 1997, a National Lymphatic Filariasis Elimination Plan was drawn up by the Ministry of Health of Brazil, creating local programs for the elimination of Bancroftian filariasis in areas with active transmission. Based on a comprehensive bibliographic search for available studies and reports of filariasis epidemiology in Brazil, current status of this parasitic infection and the outlook for its elimination in the country were analysed. From 1951 to 1958 a nationwide epidemiological study conducted in Brazil confirmed autochthonous transmission of Bancroftian filariasis in 11 cities of the country. Control measures led to a decline in parasite rates, and in the 1980s only the cities of Belém in the Amazonian region (Northern region) and Recife (Northeastern region) were considered to be endemic. In the 1990s, foci of active transmission of LF were also described in the cities of Maceió, Olinda, Jaboatão dos Guararapes, and Paulista, all in the Northeastern coast of Brazil. Data provide evidence for the absence of microfilaremic subjects and infected mosquitoes in Belém, Salvador and Maceió in the past few years, attesting to the effectiveness of the measures adopted in these cities. Currently, lymphatic filariasis is a public health problem in Brazil only in four cities of the metropolitan Recife region (Northeastern coast). Efforts are being concentrated in these areas, with a view to eliminating the disease in the country.

## Review

### Background

Lymphatic filariasis (LF) is a debilitating disease with serious social and economic impact. Marked by a wide range of clinical manifestations in humans, it is among the so-called neglected tropical diseases and is more common in disadvantaged populations lacking sanitation services and treated water supply. According to the World Health Organization (WHO), the infection is endemic in 72 countries in Asia, Africa and the Americas, with the number of affected people estimated at 120 million, being 108 million of them being infected with *Wuchereria bancrofti* and 12 million with *Brugia malayi* or *B. timori*[1,2]. In the Americas region, the disease is caused exclusively by *W. bancrofti*, with active transmission in Haiti, the Dominican Republic, Guiana, and Brazil [1,3].

Since 1997, when Resolution WHA 50.29 of the World Health Assembly set the year 2020 as the target for LF elimination as a global public health problem, the WHO has been engaged to meet this objective [4,5].

The Global Programme to Eliminate Lymphatic Filariasis (GPELF) was launched in 2000 with the objective to eliminate the disease as a public health problem by 2020 [2]. This programme has adopted a strategy consisting of the following components: (i) to stop the spread of infection (interrupting transmission); and (ii) to alleviate the suffering of affected populations (controlling morbidity).

This paper reviews the epidemiological studies on LF in Brazil, explores the current status of this parasitic disease and the outlook for its elimination in the country.

### Review methodology

A comprehensive electronic search for available data of LF epidemiology in Brazil was performed. Publications were captured from PubMed and Scielo (Scientific Electronic Library Online) databases. Either one of the following keywords written in English or in Portuguese were used: filariasis, Bancroftosis, lymphatic filariasis, *Wuchereria bancrofti*, combined to form a phrase with epidemiology, control, elimination, survey and Brazil. The abstracts of the retrieved articles were reviewed, and if they did not explicitly show the surveyed population, diagnostic method, sample size and prevalence, they were excluded. Another search strategy adopted involved bibliographic investigation in library collections of any other type of literature sources, including university theses, unpublished surveys, old journals of Health from Brazil, and reports of the Ministry of Health and WHO, in order to retrieve studies and reports on filariasis distribution and control in the country.

### Lymphatic filariasis in Brazil: past

**Figure 1:**
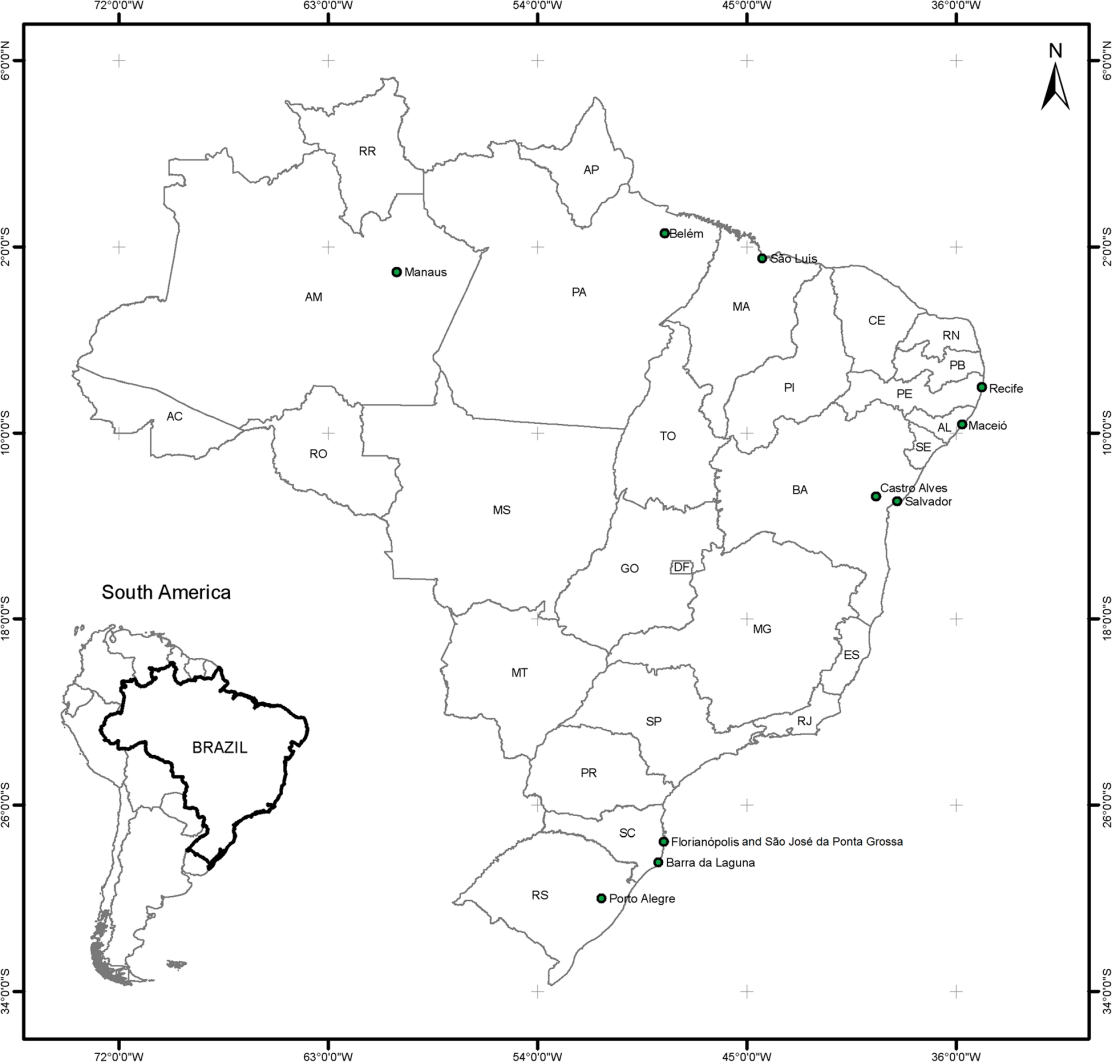
Map of geographic distribution of Lymphatic Filariasis in Brazil in the past (1950–1960).

Otto Wucherer described the first *W. bancrofti* microfilaria in Brazil in 1866, nevertheless, the first systematic epidemiological studies on the distribution of Bancroftian filariasis in this country were conducted decades later. Studies from 1940 to 1950 in Belém (Northern region) and Recife (Northeastern region) yielded microfilaremia rates of 10.8% and 9.2%, respectively, characterizing these cities as endemic areas [6,7]. These data led the Ministry of Health of Brazil to launch its National Lymphatic Filariasis Campaign. From 1951 to 1958 a total of 811,361 people were examined, using thick blood film collected nocturnally, and 120,399 specimens of mosquito vectors were dissected [8]. The simultaneous discovery of microfilaremic subjects and infected mosquitoes provided evidence of local LF transmission in 11 cities in different States, with the following prevalences: São José da Ponta Grossa (Santa Catarina State), 14.5%; Belém (Pará), 9.8%; Barra de Laguna (Santa Catarina), 9.4%; Recife (Pernambuco), 6.9%; Castro Alves (Bahia), 5.9%; Florianópolis (Santa Catarina), 1.4%; São Luis, (Maranhão), 0.6%; Salvador (Bahia), 0.4%; Maceió (Alagoas), 0.3%; Manaus (Amazonas), 0.2%; and Porto Alegre (Rio Grande do Sul), 0.1% [8] (Figure 1).

Bancroftian filariasis in Brazil was an urban phenomenon with focal characteristics and found mainly along the coast. Belém and Recife were considered the cities of greatest epidemiological importance due to their high population density and vector prevalence and density [9].

The National Lymphatic Filariasis Campaign strategy in the 1950s was based on treatment of individually diagnosed patients with diethylcarbamazine [10]. Nearly all foci were considered extinguished, and in the 1980s the Ministry of Health regarded only the cities of Belém and Recife as active areas of transmission in Brazil [11].

### Lymphatic filariasis in Brazil: interruption of transmission in historical foci

Since the national study in the 1950s, no survey has been conducted to update the information on the geographic distribution of LF all over the country, and the available data were from isolated studies. The entomological surveys conducted since 1990s are summarized in Table 1.

**Table 1:** **Entomological studies conducted to survey *Culex quinquefasciatus *females harboring infective *Wuchereria bancrofti *larvae in historical lymphatic filariasis foci in Brazil**

**City/State**	**Local**	Number of examined*	**Number of females with larvae L**_**3**_**(%)**	**Reference**
Recife/PE	Neighbours of parasitized individuals***	7,856	48 (0.61)	[12]
Olinda/ PE	Neighbours of parasitized individuals***	8,003	105 (1.31)	[12]
Jaboatão de Guararapes/PE	Neighbours of parasitized individuals***	8,010	97 (1.21)	[12]
Maceió/AL	Feitosa district	1,321	28 (2.1)	[13]
Maceió/AL	Jacintinho district	529	2 (0.4)	[13]
Maceió/AL	House of parasitized individuals-Feitosa district	467	16 (3.4)	[14]
Maceió/AL	Neighbours of parasitized individuals-Feitosa district***	1,426	21 (1.5)	[14]
Maceió/AL	House of parasitized individuals- Jacintinho district	564	6 (1.1)	[14]
Maceió/AL	Neighbours of parasitized individuals-Jacintinho***	1,403	4 (0.3)	[14]
Maceió/AL	House of parasitized individuals-endemic area	675	1 (0.15)	[15]
Maceió/AL	Parasitized neighbours-endemic area***	1,802	1 (0.055)	[15]
São José Ponta Grossa/SC	Random localities	624	0 (0.0)	[16]
Belém/ PA	57 districts	24,463	0 (0.0)	[17]
Belém/ PA	31 historically endemic districts	26,400**	0 (0,0)	[18]
Salvador/BA	22 historically endemic districts	23,580**	0 (0,0)	[19]

* Females examined by dissection and microscopy or ** by Polymerase Chain Reaction (PCR).

*** Neighbouring dwellings at 20 meters to the left or right of microfilaremic carriers houses.

Cases of filariasis infection in Salvador city registered in 1954 were distinguished by clustering of the disease in Uruguai and São Domingos districts [8]. Since then, few studies were conducted to monitor prevalence rates. A city survey carried out in 1958, revealed a prevalence of 0.09%. In 1966, Uruguai district remained the main endemic area in Salvador with 6.2% microfilaremia rate [20]. The focus has been considered extinguished with no evidence confirming that transmission of the disease has been eliminated in the city [11]. No reports were found on the control measures or epidemiological surveillance activities undertaken to evaluate the elimination of filariasis transmission in the city, unless selective treatment of infected people at the time. Recently, from 2005 to 2009, parasitological, serological, and entomological surveys were conducted in Salvador city to update information on the prevalence of this infection. Night bleed 60 μl thick blood smear from 7,588 students were tested for microfilaria, and antigen prevalence was assayed in blood samples from 517 children (6–10 years), all were found to be negative [19]. Molecular xenomonitoring to detect filarial DNA in 23,580 female mosquitoes vectors gave negative results. These data meet the current available criteria for absence of transmission in the area. Screening of circulating filarial antigen in blood using rapid immunochromatographic card testing (ICT), and polymerase chain reaction (PCR) for detection of filarial DNA in mosquito vectors, are tools recommended to monitor interruption of *W. bancrofti* transmission [21,22].

In Belém, Northern Brazil, the historical trend in the prevalence of the disease since the first reported cases has been consistently downward. Microfilaria prevalence rates, assessed by thick smears, decreased from 8.2% in the 1950s to 2.6%, 0.7%, 0.16% and 0.02% respectively, over the next 4 decades [17]. Between 1999 and 2004, no autochthonous case was diagnosed among 612,679 people examined across the city [17] (Table 2).

**Table 2:** Microscopic blood analysis using night thick blood smears, in the districts of urban Belém, Northern region of Brazil, 1990–2004

**Local**	**Year**	**Number of individuals examined**	**Number of microfilaremics (%)**	**Reference**
Greater Belém	1990-1994	1,062,945	180 (0.017)	[17]
6 Districts	1995	226,796	6 (0.002)	[17]
5 Districts	1996	93,498	15 (0.016)	[17]
4 Districts	1997	133,198	6 (0.004)	[17]
5 Districts	1998	115,279	1 (0.0008)	[17]
7 Districts	1999	152,255	0 (0.0)	[17]
6 Districts	2000	132,388	0 (0.0)	[17]
15 Districts	2001	99,093	1 (0.001)*	[17]
21 Districts	2002	92,463	0 (0.0)	[17]
12 Districts	2003	71,555	0 (0.0)	[17]
31 historically endemic districts	2004	164,018	0 (0.0)	[18]

* Case of a migrant from a historically endemic area.

In evaluating LF elimination programs in endemic countries, an area is considered transmission-free when antigenemia, determined by ICT, is less than 0.1% in a sample of 3,000 children aged 6 to 10 years [5]. In 2003, using the ICT, a study was conducted in Belém on a random sample of 3,000 students aged 6 to10 years old in areas historically endemic for Bancroftian filariasis, all tests were antigen-negative [23]. In 2004, another study was conducted in the same area among 2,816 men between 20 to 30-years old, the most susceptible population to the infection, with ICT negative results [24].

From 2002 to 2004, vectors were captured in 57 out of the 71 districts of Belém. *C. quinquefasciatus* mosquitoes were examined by dissection (24,463) or PCR (26,400), in the same areas where 164,018 individuals had been tested using the thick blood film technique (50–60 μL blood) [17]. No microfilaremic subjects or infected mosquitoes were found [18]. The data confirm the lack of active transmission in Belém, the only focus of Bancroftian filariasis in the Northern region of Brazil.

In Santa Catarina State in the Southern region, in a 1976 study involving microscopic thick blood analysis, 21,639 residents in the three areas previously identified as endemic were tested, with no positive results [25]. In order to verify elimination of the disease in these foci, 206 residents of São José da Ponta Grossa (90.7% of the population) and 1,154 of Barra de Laguna (95.2% of the population) were examined and no cases were detected in areas with prevalences of up to 14.5% in the 1950s [26]. In these same locations 38 treated individuals, diagnosed microfilaremic in the 1950s, were revaluated by different techniques with negative results. Furthermore, none of the 624 captured and dissected *C. quinquefasciatus* mosquitoes harboured *W. bancrofti* larvae [16]. This data indicates that there is no longer active transmission of Bancroftian filariasis in the historical foci of Santa Catarina.

In Maceió, Alagoas State in Northeastern Brazil, the National Lymphatic Filariasis Campaign of the 1950s, when the prevalence was 0.3% [27], confined its efforts to treatment of parasitized individuals. Since then, filariasis has been considered as extinguished in the city [11]. However, the discovery of three autochthonous microfilaremic individuals in 1990 [28] triggered a broad epidemiological study in Maceió. A cross-sectional study was conducted and a total of 10,857 night students from every district in the city were evaluated by thick blood smears, with 73 (0.7%) microfilaremics detected [29]. Distribution of the disease proved to be focal, with the infected people concentrated in three central adjacent districts in the city, with prevalences ranging from 1.2 to 5.3% [29]. In these locations, the captured *C. quinquefasciatus* mosquitoes exhibited infection rates from 0.4 to 2.1% [13] (Table 1). Another study was conducted to assess the distribution of filariasis in the general population in Maceió and nine cities in the different physiographic regions of Alagoas State [30]. In Maceió microfilaria status of 10,973 individuals from all age groups were determined through examination of thick smears made from approximately 60 μL of finger prick blood, in the three endemic districts the average prevalence of microfilaremics was 2.5%. In the others cities studied no autochthonous cases were found among the 20,103 people tested [30]. However, with the growing migration in the country, there is a risk that the disease might be introduced in areas free of the infection. In Sri Lanka infected migrants have engendered LF in areas where the disease was previously unknown [31]. This occurred also in metropolitan Recife, where cases of the parasitosis appeared in previously unaffected areas [32,33].

In Maceió, in the early 2000s, the natural infection rates of mosquitoes captured in the houses of parasitized individuals and neighbouring houses were 0.15% and 0.055%, respectively [15]. These rates were lower than those previously obtained in a similar study in the same region [14] (Table 1).

Since the launch of the Lymphatic Filariasis Elimination Program in Maceió in 1999, the population in the endemic area has been continuously monitored to detect and treat parasitized individuals. A complementary antigen study of 3,000 children in 2003, using ICT observing the WHO criteria [5], yielded 10 (0.3%) antigen-positive cases. Despite its low antigenemia, Maceió at that time still had the potential for transmitting Bancroftian filariasis [3].

Epidemiological surveillance in Maceió’s endemic area, using 50–60 μL thick blood smears, from 1999 to 2005, has revealed a substantial decline in the frequency of microfilaremia, as follows: 0.74% (1999); 0.54% (2000); 0.49% (2001); 0.10% (2002); 0.08% (2003); 0.06% (2004) and 0.0% (2005) [34]. The results of blood analysis in Maceió and other cities in Alagoas State from 1990 to 2005 are presented in Table 3.

**Table 3:** Microscopic analysis using night thick blood smears in Maceió and others cities of Alagoas State, Northeastern region of Brazil, 1990–2005

**City**	**Local**	**Number of individuals examined**	**Number of microfilaremics (%)**	**Reference**
Maceió	Army Military Command	731	2 (0.3)	[28]
Maceió	Students - 33 districts	10,857	73 (0.7)	[29]
Paripueira	Urban area	2,205	2 (0.09)*	[30]
Maragogi	Urban area	1,795	0 (0.0)	[30]
Porto Calvo	Urban area	2,989	0 (0.0)	[30]
Marechal Deodoro	Urban area	2,765	0 (0.0)	[30]
Coruripe	Urban area	2,479	0 (0.0)	[30]
Palmeira dos Índios	Urban area	2,149	0 (0.0)	[30]
Maribondo	Urban area	1,975	0 (0.0)	[30]
Piranhas	Urban area	789	0 (0.0)	[30]
Pão de Açúcar	Urban area	2,957	0 (0.0)	[30]
Maceió	Feitosa district	2,450	133 (5.4)	[30]
Maceió	Pitanguinha district	1,865	43 (2.3)	[30]
Maceió	Jacintinho district	4,637	50 (1.1)	[30]
Maceió	Chã da Jaqueira district	2,021	0 (0.0)	[30]
Maceió	Endemic area (1999)	2,821	21 (0.74)	[34]
Maceió	Endemic area (2000)	12,669	69 (0.54)	[34]
Maceió	Endemic area (2001)	13,544	66 (0.49)	[34]
Maceió	Endemic area (2002)	24,159	23 (0.10)	[34]
Maceió	Endemic area (2003)	7,450	6 (0.08)	[34]
Maceió	Endemic area (2004)	6,715	4 (0.06)	[34]
Maceió	Endemic area (2005)	9,425	0 (0.0)	[34]

* Non-authochonous cases.

Since 1990, when systematic and uninterrupted study of LF in Maceió began, 2005 was the first year with no new microfilaremics identified, even when testing 9,425 individuals in the endemic area and its surroundings through thick blood film. Corroborating these data, a cross-sectional survey was performed in 2007 in a random sample of 20,024 night students from 143 schools in the 50 city districts, with no positive exam. From 2005 to 2007, none of the 2,583 vectors examined by PCR were found to be infected [35]. In 2009 an antigenemia research using ICT in 3,000 children was carried out with no positive results. The steps taken have lead to a significant reduction in the number of parasitized individuals to a point where no microfilaremic or infected mosquito could be detected in the past seven years in Maceió, indicating a possible elimination of LF transmission in the city.

### Lymphatic filariasis in Brazil: active foci in the present

In Brazil, filariasis continues to be of significant local importance in Pernambuco State, particularly in Recife (capital of the State) and its metropolitan areas, Olinda, Jaboatão dos Guararapes and Paulista (Table 4) [3,33,36-39]. Entomological studies showed *C. quinquefasciatus* infection rates of 0.6% in Recife and over 1.0% in the cities of Olinda and Jaboatão dos Guararapes [12] (Table 1). These data indicate that active LF transmission occurred in Recife, and in cities of the metropolitan region. The current program to interrupt transmission of LF in these areas, which comprises 0.8% of the national population, is based on mass drug administration (MDA), successfully used to control filariasis in some African and Asian endemic countries [40].

**Table 4:** Microscopic analysis of night thick blood smears, performed in cities of metropolitan Recife, Northeastern region of Brazil, 1990–2011

**City**	**Local**	**Number of individuals examined**	**Number of microfilaremics (%)**	**Reference**
Recife Metropolitan Region	Army Military Command	23,773	582 (2.5)	[32]
Recife	St. Amaro/Campo Grande	466	63 (13.5)	[41]
Recife	Coque/Mustardinha distritcs (children)	1,464	93 (6.4)	[42]
Recife	Coque/Mustardinha districts	4,597	460 (10.0)	[43]
Recife	31 districts	10,581	683 (6.5)	[44]
Olinda	Sapucaia/Salgadinho districts	685	84 (12.3)	[41]
Olinda	Azeitona district	541	56 (10.3)	[45]
Olinda	Urban area	5,258	328 (6.2)	[37]
Jaboatão Guararapes	Cavaleiro district	9,520	213 (2.2)	[36]
Jaboatão Guararapes	Urban area	4,367	33 (0.8)	[38]
Jaboatão Guararapes	Urban area	23,673	323 (1.4)	[39]
Jaboatão Guararapes	Urban area	8,670	96 (1.1)	[46]
Moreno	Urban area	2,513	2 (0.08) *	[47]
Cabo St. Agostinho	Urban area	7,650	6 (0.08)*	[33]
Paulista	Urban area	25,526	55 (0.22)	[3]

* Non-autochthonous cases.

Before introduction of control measures blood samples taken for direct parasitological examination (thick blood film) of a sample of 10,581 individuals from 31 districts of Recife, resulted in 6.5% infected carriers, with district prevalences ranging from 0.0 to 14.6% [44]. In two of these districts, Coque and Mustardinha, examination of 45 μL peripheral blood of 5,563 subjects (5 to 65 years) revealed a microfilaria prevalence rate of 10% [43]. In the same districts, a study of the pediatric population (5 to 14 years), using the same technique showed 6.4% microfilaria carriers [42].

In a larger parasitological survey conducted with 23,773 Brazilian soldiers from metropolitan Recife, 60 μL of blood were examined for microfilaria. A total of 582 (2.5%) of those found to be infected were from areas previously recognized as endemic for LF (Recife, Olinda, Jaboatão dos Guararapes) and also from areas until then considered unaffected (Abreu e Lima, Cabo de Santo Agostinho and Paulista) [32].

In order to map the distribution of LF in metropolitan Recife studies were also conducted in Moreno, Cabo de Santo Agostinho and Paulista, cities surrounded by areas of LF transmission. In Moreno, a rate of 0.08% microfilaremia was found, but none of the cases were autochthonous, indicating that filariasis transmission is not a problem [47]. In Cabo de Santo Agostinho, in the late 1990s, an autochthonous case of LF was found. More recently, 0.08% of the 7,650 examined people had microfilaremia; one of these cases was proven to be autochthonous, indicating the potential for LF transmission in this area [33]. In 2002, microscopic blood analysis in the city of Paulista yielded a 0.22% microfilaremia rate [3]. This data shows that disease has spread to new places probably due to the migration of infected people.

Before MDA intervention different districts of Olinda showed prevalence rates from 6.2 to 12.3% [37,41,45].

In Jaboatão dos Guararapes, recently a mean prevalence rate of filarial infection of 2.2% was observed using thick blood smears examination [36]. Antigen studies in the population aged 19 and below, using the ICT, yielded a 5.5% positive rate [48]. In this same city 85.7% of the neighbourhoods presented positive microfilaria carriers, detected by finger prick blood examination, with prevalences ranging from 0.1 to 2.9% (mean=0.8%). Prior to MDA, in the municipality of Jaboatão dos Guararapes, examination of 50 μL of capillary blood of 8,670 children and adolescents (6–14 years old) revealed 96 microfilaria carriers (1.1%) and the spatial distribution of microfilaremia showed that 13 (54.2%) of the 24 districts investigated were positive [46]. Another study showed 323 (1.4%) individuals were infected among 23,673 of those diagnosed by night thick blood smears. Prevalences up to 25% stratified by census tracts and analyzed according to socio-environmental and social deprivation indexes, indicated that the greatest risk of filariasis transmission is found in the high-risk strata [39].

Antifilarial MDA, the main strategy recommended by WHO for Lymphatic Filariasis elimination, was launched in Recife in 2003. It involved at first 18,087 people in peripheral population, treated with a single dose of diethylcarbamazine citrate, and had been scaled up in the following years to 141,528 people in 2009, at a financial cost ranging from U$ 0.42 to U$ 0.78/person treated [49,50].

The MDA intervention in the municipality of Olinda started in 2005 and until 2010 43,695 inhabitants were treated [49,51]. A survey conducted in 2007 using ICT detected 66 (9.8%) antigen positive individuals out of 672 school children, indicating active transmission in the evaluated area [52]. Based on these data the Municipal Health Secretary of Olinda expanded MDA, and is making efforts to strengthen health education, social mobilization, morbidity management, and vector control measures [50,51].

According to the document from the Regional Program Manager’s Meeting, additional vector control measures in Recife and Olinda are helping to decrease LF prevalence [50].

Jaboatão dos Guararapes has been under MDA program since 2006, but no reports of the post treatment LF prevalence is available.

Of the last four foci describing LF in Brazil, MDA was not introduced only in the municipality of Paulista, because prevalence decreased from 0.25% in 2002 to 0.008% in 2010 [49].

### Remarks

In 1997, responding to the resolution of Brazil’s National Health Council (Resolution No.190 of 13/06/1996) and WHO’s call for the Global Elimination of Lymphatic Filariasis, the Ministry of Health drew up the National Lymphatic Filariasis Elimination Plan (PNEFL) [53]. The main objectives of the PNEFL are: epidemiological reassessment of active foci as well as those considered extinguished; community mobilization; interruption of LF transmission in the endemic foci through specific treatment and vector control; and prevention and reducing disability in affected persons.

With the launch of the PNEFL in Brazil, local programs for the elimination of Bancroftian filariasis were created or implemented in areas of active transmission. Control measures implemented in each endemic area are listed in Table 5.

**Table 5:** Intervention and surveillance activities carried out in lymphatic filariasis endemic areas in Brazil

**City/State**	**Intervention activities**
Maceió/Alagoas	- Training of field and health personnel
	- Case-finding (active and passive by microscopic blood examination)
	- Determination of microfilaria carriers prevalence (baseline)
	- Mapping of endemic foci
	- Identification of the population at risk of infection
	- Entomological survey
	- Selective treatment of microfilaria carriers
	- Patients follow up (at least 2 years)
	- Medical care to control morbidity
	- Antigen test for diagnosis of Bancroftian filariasis in children by ICT
	- Xenomonitoring (detection of filarial DNA in mosquitoes by PCR)
	- Surveillance (haematological/serological and entomological)
Belém/Pará	- Training of field and health personnel
	- Case-finding (active and passive by microscopic blood examination)
	- Determination of microfilaria carriers prevalence (baseline)
	- Mapping of endemic foci
	- Identification of the population at risk of infection
	- Entomological survey
	- Selective treatment of microfilaria carriers
	- Medical care to control morbidity
	- Improvement of environmental sanitation
	- Antigen test for diagnosis of Bancroftian filariasis in children by ICT
	- Xenomonitoring (detection of filarial DNA in mosquitoes by PCR)
	- Surveillance (haematological/serological and entomological)
Salvador/Bahia	- Training of field and health personnel
	- Determination of microfilaria carriers prevalence (baseline)
	- Mapping of endemic foci
	- Identification of the population at risk of infection
	- Selective treatment of microfilaria carriers
	- Antigen test for diagnosis of Bancroftian filariasis in children by ICT
	- Xenomonitoring (detection of filarial DNA in mosquitoes by PCR)
	- Surveillance (haematological/serological and entomological)
São José Ponta Grossa/ Santa Catarina	- Case-finding (active and passive by microscopic blood examination)
	- Determination of microfilaria carriers prevalence (baseline)
	- Mapping of endemic foci
	- Identification of the population at risk of infection
	- Entomological survey
	- Selective treatment of microfilaria carriers
	- Antigen test for diagnosis of Bancroftian filariasis in children by ICT
	- Surveillance (haematological/serological and entomological)
Recife and its metropolitan region	- Training of field and health personnel
	- Case-finding (active and passive by microscopic blood examination)
	- Determination of microfilaria carriers prevalence (baseline)
	- Mapping of endemic foci
	- Identification of the population at risk of infection
	- Entomological survey
	- Selective treatment of microfilaria carriers
	- Mass drug administration (MDA) since 2003
	- Medical care to control morbidity
	- Management of morbidity (lymphedema treatment)
	- Social support (Hope clubs)
	- Information to generate community awareness and social mobilization
	- Integrated mosquito control measures
	- Antigen test for diagnosis of Bancroftian filariasis in children by ICT
	- Xenomonitoring (detection of filarial DNA in mosquitoes by PCR)
	- Surveillance (haematological/serological and entomological)

Endemic areas detected in the 1950s and not subsequently evaluated are under evaluation to assess if LF has been eliminated in these localities.

From the second half of the 1990s Belém, which had the highest prevalences of LF, have been the object of sweeping environmental sanitation efforts involving the drainage of canals, relocation of wetland populations, and restoration of degraded areas through landfills, tree-planting, and paving.

In Maceió, selective treatment of parasitized individuals and their follow up for at least two years in order to confirm the clearance of microfilaremia, led to elimination of the sources of vector infection since 2004. The data from Belém and Maceió confirm the importance of ensuring the continuity of LF control and elimination programs.

The traditional intervention strategy in Brazil has consisted chiefly of microscopic blood analysis, selective treatment, and care for cases of filarial morbidity. Since 2003, MDA with diethylcarbamazine was undertaken in a sample of the population in endemic areas of Recife and cities of its metropolitan region [54]. The population selected for MDA consisted of residents in LF foci in Recife, Olinda and Jaboatão. In 2003, 18,000 treatments/year were administered, covering a population of 23,400 people [54]. Annual MDA coverage was reported to be 87% of the 63,800 people who qualified for mass treatment in these areas [54]. To scale up MDA in 2010, 154,056 treatments/year were administered, covering 76.5% of the population out of 201,385 eligible people [2].

Parasitological and entomological surveillance during the past few years has demonstrated that the transmission of Bancroftian filariasis has been interrupted in Belém, Maceió, Salvador and the three historical foci of Santa Catarina, with no cases found either by microscopic analysis and/or antigenemia assessment. Furthermore, in Belém and Maceió, entomological surveys reported that none of the vector were infected with *Wuchereri*a. Therefore, in Brazil efforts to eliminate the disease should be concentrated in the remaining active foci of LF transmission in metropolitan Recife, freeing Brazil of Bancroftian filariasis according of WHO’s global target. Interventions like MDA, vector control, and other actions to control LF must continue in this area to guarantee a steady reduction in endemicity. Considering the biological characteristics of the local vectors, integrated strategy involving MDA and vector control measures could accelerate parasite elimination, reducing the number of years necessary to interrupt parasite transmission. This strategy is more effective in areas where transmission depends on *Anopheles* instead of *Culex* species, but in either case helps the sustainability of filariasis transmission interruption [55].

Because of the lack of a gold standard test it is difficult to demonstrate with certainty the interruption of *W. bancrofti* transmission. However, it is enough to achieve a threshold prevalence below which the parasite transmission is not sustained [21,22].

## Conclusion

Knowledge of the current LF situation in Brazil is essential for implementing joint programs for the elimination of the disease and for setting up epidemiological surveillance programs in locations where microfilaremics are no longer found, to prevent the emergence of new foci and/or reemergence of the disease in areas where it has been eliminated.

Success of the PNEFL and interruption of transmission will prevent the appearance of morbidity such as elephantiasis, hydrocele, and other clinical forms of the disease, as well as its resulting psychological, social, and economic impact.

## Competing interests

The authors declare that they have no competing interests.

## Authors’ contributions

GF conceived the idea for the review, analysed data, wrote the initial draft and finalized the manuscript. EMMR analysed data, wrote the initial draft and finalized the manuscript. ABL and ARVL analysed data. HF analysed data and provided critical comments. JPE intellectually supported the study and corrected the drafts of the manuscript. All authors approved the final version of the manuscript.
